# A Deep Learning Approach for Predicting Antigenic Variation of Influenza A H3N2

**DOI:** 10.1155/2021/9997669

**Published:** 2021-10-16

**Authors:** Yuan-Ling Xia, Weihua Li, Yongping Li, Xing-Lai Ji, Yun-Xin Fu, Shu-Qun Liu

**Affiliations:** ^1^State Key Laboratory for Conservation and Utilization of Bio-Resources in Yunnan, Yunnan University, Kunming 650091, China; ^2^Editorial Office of Journal of Yunnan University (Natural Sciences Edition), Yunnan University, Kunming 650091, China; ^3^School of Information Science and Engineering, Yunnan University, Kunming 650091, China; ^4^School of Agriculture, Yunnan University, Kunming 650091, China; ^5^Smart Health Big Data Analysis and Location Services Engineering Lab of Jiangsu Province, Nanjing University of Posts and Telecommunications, Nanjing 210023, China; ^6^Human Genetics Center and Division of Biostatistics, School of Public Health, The University of Texas Health Science Center, Houston, TX 77030, USA

## Abstract

Modeling antigenic variation in influenza (flu) virus A H3N2 using amino acid sequences is a promising approach for improving the prediction accuracy of immune efficacy of vaccines and increasing the efficiency of vaccine screening. Antigenic drift and antigenic jump/shift, which arise from the accumulation of mutations with small or moderate effects and from a major, abrupt change with large effects on the surface antigen hemagglutinin (HA), respectively, are two types of antigenic variation that facilitate immune evasion of flu virus A and make it challenging to predict the antigenic properties of new viral strains. Despite considerable progress in modeling antigenic variation based on the amino acid sequences, few studies focus on the deep learning framework which could be most suitable to be applied to this task. Here, we propose a novel deep learning approach that incorporates a convolutional neural network (CNN) and bidirectional long-short-term memory (BLSTM) neural network to predict antigenic variation. In this approach, CNN extracts the complex local contexts of amino acids while the BLSTM neural network captures the long-distance sequence information. When compared to the existing methods, our deep learning approach achieves the overall highest prediction performance on the validation dataset, and more encouragingly, it achieves prediction agreements of 99.20% and 96.46% for the strains in the forthcoming year and in the next two years included in an existing set of chronological amino acid sequences, respectively. These results indicate that our deep learning approach is promising to be applied to antigenic variation prediction of flu virus A H3N2.

## 1. Introduction

Influenza (flu) A virus poses a persistent threat to global public health because it causes not only the seasonal epidemics of flu disease but also the global flu pandemic. Even the less deadly seasonal epidemics alone accounted for approximately 24000 deaths in the USA annually from 1976 to 2007 [1], and the latest report estimates that the number of flu deaths increases to 61000 in 2017-2018 flu season [2, 3]. It is now known that the homotrimeric surface glycoprotein hemagglutinin (HA) is responsible for binding the virus to the host cell surface receptor which leads to virus entry [4]; HA is thus the primary antigen targeted by the host immune system [5]. Although there is another glycoprotein neuraminidase (NA) found on the surface of flu viruses, NA is generally considered less important in terms of the antigenicity than HA [6]. The HA protein is synthesized as a single-chain polypeptide precursor, HA0, which is subsequently cleaved into two subunits (HA1 and HA2) that form a homotrimeric spike on the virus surface [7]. Compared to HA2, the HA1 subunit mutates more frequently and faces a stronger selection pressure from the host immune system, ultimately resulting in the emergence of the immune-evading variants/strains [8]. Preparation of the flu vaccine that comprises the viral immunogens capable of eliciting neutralizing antibodies against the virus strains most likely to circulate in the forthcoming flu season is currently the most effective means of preventing flu infection [9, 10]. However, two kinds of antigenic variation, the antigenic drift and antigenic shift/jump arising from the rapid evolution of HA, allow flu viruses to escape host immunity [11]; this presents continuous challenges for the selection of the vaccine strains to be matched. Since 1977, the flu A subtypes of H1N1 and H3N2 and the flu B have been circulating globally and hence are prior strains included in the vaccine program [12]. Of note is that the flu A H3N2 is the most common subtype causing human infection and disease in the past 40 years [13].

For a forthcoming flu pandemic or any other newly emerging flu strain, it is important to predict the antigenic property of the causative virus so as to prepare the effective vaccine. The degree of antigenic similarity in the pairwise flu strains is examined mainly by the hemagglutination inhibition (HI) test [14, 15] in laboratory. However, the serology assay, a key step in the HI test, is very time-consuming and labor-intensive. Consequently, the HI test for newly emerging strains is severely lagging behind the rapid accumulation of new strains that spread globally. For example, the numbers of HA sequences of the H3N2 human flu virus submitted to the NCBI influenza virus database in 2014, 2015, and 2016 were 1959, 2229, and 1735, respectively, which would require more than 70 million pairwise comparisons for thoroughly determining antigenic variation by the HI test, an obviously unrealistic task. Indeed, at present, there are only sparse reports of HI tests in limited literature [16]. Since the virus genomes are routinely examined by high-throughput sequencing, sequence comparison has been providing extremely valuable information on variations in the antigenicity of flu strains, which will help to surveil the emergence of novel variants, reduce the detection time of new antigenic types, and improve the efficiency of vaccine development and preparation.

The last 15 years have witnessed a considerable progress in predicting the antigenic variation of flu viruses based on the HA1 amino acid sequence. Lee and Chen made a pioneering attempt [17] to model the antigenic variation using a simple binary indicator to identity whether or not the number of amino acid mutations exceeds a threshold value while the prediction results were not satisfactory. Realizing the nonequivalent importance of the 329 HA1 amino acid residues in determining the antigenicity, Liao et al. [18] predicted the contribution of 19-23 selected amino acid positions to antigenic variation through amino acid classification and multiple regression analysis, with the results showing reasonable prediction sensitivity but poor specificity. Huang et al. [19] improved the agreement over Liao's method by constructing a decision tree based on the 19 key amino acid positions selected according to the criteria of information gain and entropy. Recognizing that different amino acid substitutions can have distinct effects on the antigenicity of HA1, Cui et al. [20] proposed a linear regression-based method where 18 key residue positions were selected by a significance score, and at each position, the effects of amino acid substitutions on the antigenic property were indicated by 8 physicochemical properties. Based on a dataset spanning from 1968 to 2007, Sun et al. [21] selected 39 key positions with bootstrapped ridge regression and quantitatively measured the antigenic distances through antigenic mapping [22]; although the accuracies of prediction for the next flu seasons were high, such accuracies might have been inflated since the 39 key positions were derived from analyzing the whole dataset that includes the validation set. Through combining multiple feature matrices derived from different amino acid similarity matrices to construct decision trees in a random forest algorithm, Yao et al. [23] proposed a joint random forest regression (JRFR) method to predict antigenic distances from HA1 sequence data, with the 10-fold cross-validation results showing that JRFR outperforms other popular methods in predicting antigenic variants.

Essentially all the aforementioned methods have three characteristic key steps. The first step is to identify amino acid residues (key positions) that likely contribute to antigenic variation using a subset of available data (training set), the second step is to model the relationship between antigenic variation and these key positions using the training set, and the third step is to apply the derived model to both the training set and validation set to determine the accuracy of the method.

Although the key-position-based prediction methods have achieved great success, they often fail to extract complex nonlinear relationships from the entire HA1 sequence. Fortunately, the recently popular deep learning techniques are advantageous in automatically representing the original sequence and learning the hidden patterns through nonlinear transformations and hence are very suitable for the prediction of the antigenic property based on the amino acid sequence comparison. Deep learning techniques have been encouraged by their tremendous success in computer vision [24], speech recognition [25], and sentiment classification [26] and now are widely applied to many areas of biological research including protein contact maps [27], drug-target binding affinity [28], regulatory network [29], and protein features [30, 31]. Recently, Tan et al. [32] employed the stacked autoencoder (SAE) model to predict an antigenic variant of flu A H3N2; however, the results showed that SAE did not have a distinct advantage over the other machine learning algorithms.

In this paper, we introduce a deep learning approach to predict the antigenic variation of flu A H3N2 strains based on the sequence comparison of HA1 proteins. This approach incorporates convolutional neural network (CNN) and bidirectional long-short-term memory (BLSTM) neural network, which are responsible for extracting the local and nonlocal sequence information, respectively, to predict antigenic variation of flu A H3N2. The results show that our deep learning approach achieves the overall best prediction performance on the validation set as compared to the existing methods.

## 2. Methods

### 2.1. Dataset

The antigenic distance of pairwise viruses is defined as the geometric mean of two ratios between the heterologous and homologous hemagglutination inhibition titers characterized by the ferret antiserum cross reactivity [33]. Let *c*_*ij*_ be the minimum concentration of the antiserum that was induced by the flu strain *i* but can inhibit hemagglutination by the virus strain *j*; then, the antigenic distance (also known as Archetti-Horsfall distance [34]) between strains *i* and *j* is defined as $d_{ij}=\sqrt{c_{ij}c_{ji}/c_{ii}c_{jj}}$ [33]. If the value of *d*_*ij*_ is not larger than 4 [34, 35], the inactivated vaccine prepared with the strain *i* is considered effective for preventing infection by the strain *j*. According to the Archetti-Horsfall distance definition, every antigenic distance *d*_*ij*_ should be derived from four HI tests, which slows down the progress of vaccine preparation/development.

The above measurement is rarely used directly to determine the antigenic distance of virus pairs on a large scale due to the complexity of antiserum preparation in reality. New strains are commonly detected by the HI test using a series of standard antisera that could indicate their antigenicity. If *n* strains are tested by *m* standard antisera, one can obtain a matrix *H* with *n* × *m* elements, and the element *h*_*ij*_ is the HI response data of strain *i* and serum *j*. Due to experimental constraints, matrix *H* may contain only sparse observations of the positive response. Smith et al. [36] represented strains into two-dimensional locations using modified metric ordinal multidimensional scaling on the sparse HI matrix, thus realizing the characterization of the antigenic distance of the strain pair with Euclidean distance. Although these calculated two-dimensional positions were generally stable, there might be more than one stable state between some large subgroups. Bedford et al. [37] also proved inaccuracy in distances between strains with evolution time greater than 15 years. Smith et al. [36] clustered 253 flu A H3N2 strains into 11 classes by combining the calculated positions and known biological knowledge, whereby the obtained antigenic properties were more credible than the directly calculated distances.

The above-mentioned 11 antigenic clusters of 253 flu strains are currently the largest qualitative set of antigenic properties (hereafter referred to as Smith's dataset), although it is composed of quasi-experimental data. The dataset used in the present study was constructed based on Smith's dataset. First, the 253 virus strains in Smith's dataset were randomly assigned to the two groups with the number ratio of 7 : 3; second, the virus pairs in the first and second groups were removed according to the two criteria proposed by Du et al. [38]: (i) the paired HA1 protein sequences with more than nine antigenic variation-causing mutations and (ii) the redundant virus pairs with the same sequence vectors but different sequence names/tags; finally, we obtained a dataset consisting of 5401 virus pairs, out of which 3681 and 1720 are those composed of paired viruses with similar and altered/varied antigenicity, respectively. The reason for removing the virus pairs with more than nine antigenic variation-causing mutations is that the probability of the antigenic difference between such paired strains is 99%, thus making it unnecessary to predict antigenic variation between them [32, 38]. The virus pairs retained in the first and second groups were used as the training and validation sets, respectively, for hyperparameter tuning, feature selection, and prediction performance evaluation and comparison in the present study.

The ability of the deep learning approach to predict vaccine strains was evaluated by predicting the antigenic profiles of the strains in the forthcoming year and in the next two years based on the historical chronological data. For a given year *N* from 1991 to 1999, the training data are the strains isolated before the year *N* (from 1968 to year *N* − 1), and the validation data are the strains isolated in the year *N* or *N* + 1. The validation data in the year *N* and in the years *N* and *N* + 1 were used for predicting the antigenic variation of the strains in the forthcoming year and in the next two years, respectively. The prediction results were the sum statistics from 1991 to 1999.

### 2.2. Coding for Sequence Comparison and Key Features

Faithfully encoding the symbolic amino acid sequences of HA1 proteins of a virus pair and the features associated with the viral antigenicity is an important step for improving the performance of the deep learning approach. In this study, the raw amino acid sequence without any explicit feature engineering was used as the initial input, and each amino acid was encoded as a one-hot vector using the orthogonal encoding scheme [39]. Practically, 20 input units were assigned to describe the corresponding 20 types of amino acid residues. In the 20-dimensional space, only the digit corresponding to the rank of a residue was marked as 1 and the other 19 digits were marked as 0 (Table S1). For example, the vectors [1, 0, 0, 0, ⋯, 0, 0, 0], [0, 1, 0, 0, ⋯, 0, 0, 0], and [0, 0, 0, ⋯, 0, 0, 0, 1] represent glycine, alanine, and histidine, respectively. For the pairwise sequences, each position is represented by the vector of the corresponding logical calculation “*C*_*i*_ OR *C*_*j*_,” where *C*_*i*_ and *C*_*j*_ are vectors of the two amino acids at the same position, respectively. For example, if *C*_*i*_^*m*^ and *C*_*j*_^*m*^ are both glycine at a position *m*, the vector of the position is [1, 0, 0, 0, ⋯, 0, 0, 0] ([1, 0, 0, 0, ⋯, 0, 0, 0] OR [1, 0, 0, 0, ⋯, 0, 0, 0]); if *C*_*i*_^*m*^ and *C*_*j*_^*m*^ is glycine and histidine, respectively, the position vector is [1, 0, 0, 0, ⋯, 0, 0, 0, 1] ([1, 0, 0, 0, ⋯, 0, 0, 0] OR [0, 0, 0, ⋯, 0, 0, 0, 1]). Since the change in amino acid residue between pairwise viruses provides information crucial for assessing antigenic variation, the residue position where mutation occurs in a HA1 sequence pair was encoded as the “position” feature in our deep learning approach.

In addition to the position feature, three structure-related features, which are likely to impact the antigenicity of flu viruses, were extracted and encoded into the sequence pair to test their effects on the prediction performance of the deep learning approach. Specifically, the features named “epitope,” “RBD,” and “Gly” refer to whether or not a residue resides on the five known epitopes of H3N2 HA1 [40], belongs to the receptor-binding domain (RBD) [7], and is at the glycosylation site, respectively. The glycosylation sites of each HA1 sequence were predicted using NetNGlyc [41]. The features of epitope, RBD, and Gly for a residue at the position *m* are denoted as *E*^*m*^, *R*^*m*^, and *G*^*m*^, respectively, with their values assigned as 0.5 and 0 if the residue meets and does not meet the corresponding feature conditions, respectively. For the position equivalent residues of the paired sequences, the features of epitope and RBD are individually identical and hence are one-dimensional, while the Gly feature (*G*^*m*^) may be different and hence is two-dimensional. Finally, a feature vector matrix *A* with size of 24 × *L*:

(1) $$\begin{array}{c} A=\left[\begin{array}{c} C_{i}^{1}\;E^{1}\;G^{1}\;R^{1}\\ \vdots \;\vdots \vdots \vdots \;\\ C_{j}^{L}\;E^{L}\;G^{L}\;R^{L} \end{array}\right], \end{array}$$

where *L* is the sequence length, can be constructed as the input of the CNN.

### 2.3. Framework of the Deep Learning Approach

**Figure 1:**
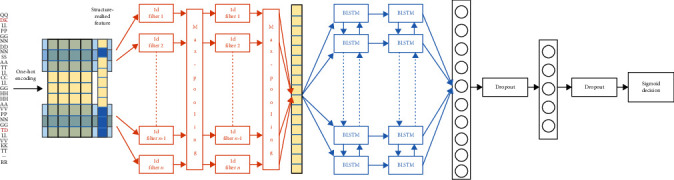
The flowchart of our deep learning approach using the one-dimensional CNN and BLSTM module.

In the 3D structure of the HA protein, there are some residues that are not close in the primary structure but are spatially close to one another. These residues were nonlocal at the sequence level, but their co-mutations could greatly affect the antigenicity of the flu virus [19]. Since the antigenic phenotype of a strain can be altered by both the local and nonlocal changes of the amino acid sequence, in our deep learning framework, the two layers, CNN and BLSTM, were used to capture information on these changes [42]. CNN, which is often applied to image recognition due to its ability of capturing the spatiotemporal feature, is also competent in capturing the local and nonlocal information on residue changes because of adjustable length of the convolution window [31, 42]. BLSTM is an artificial recurrent neural network (RNN) architecture with feedback connections, which is more advantageous in processing the entire sequence [42, 43].  Figure 1 shows the flowchart of our deep learning approach, which includes two convolutional layers connected and followed by two pooling layers and two BLSTM layers. The relevant features stored in the sequence pair are encoded into a feature vector matrix and passed to the fully connected core layers. To avoid overfitting, two dropout functions are used, with the first dropout located between the two fully connected layers and second one following a fully connected layer. Finally, the sigmoid function is used for classification. The above deep learning procedure can easily be implemented by the high-level neural network API tool, Keras (https://github.com/keras-team/keras), whose backend is TensorFlow (https://www.tensorflow.org/).

#### 2.3.1. Convolution and Pooling

The feature vector matrix *A*, which contains the one-hot encoded input features, is convolved using one-dimensional CNN with *n* convolution filters (Figure 1), with each filter *F*_*j*_ being applied to the window of *f* amino acid residues by the activation function rectified linear unit (ReLU) along the protein sequence length *L*. For each filter *F*_*j*_, the ReLU function on the windows is applied *L* times as described by

(2) $$\begin{array}{c} m_{i}=\text{ReLu}\left(F_{j}•a_{i:i+f-1}+B_{r}\right),\;i,j\in R^{L}, \end{array}$$

where • represents the dot product and *B*_*r*_ is the bias term. The feature map *m*^*j*^ of the filter *F*_*j*_ is defined by

(3) $$\begin{array}{c} m^{j}=\left[m_{1},\cdots ,m_{L}\right]. \end{array}$$

Then, the feature vector *M* = [*m*^1^; ⋯; *m*^*j*^; ⋯; *m*^*n*^] is obtained from the *n* filters.

1D max-pooling operations are performed on the vector *M* to avoid overfitting. This is described by

(4) $$\begin{array}{c} s_{i}=Pm\left(M_{i:i+q}\right),\;i\in R^{L}, \end{array}$$

where *Pm*(·) represents the 1D max-pooling function and *q* is the pool size. The whole pooling results can be indicated by

(5) $$\begin{array}{c} S=\left[s_{1},\cdots ,s_{L/q+1}\right]. \end{array}$$

#### 2.3.2. Long-Short-Term Memory Networks

The above 2D data of (*L*/*q* + 1) × *n* is then flatted to 1D data of 1 × (*L*/*q* + 1) × *n* suitable for the LSTM layer. In the deep learning framework, the bidirectional LSTM layer is aimed at extracting the long information from the pseudo sequence comparison. The basic unit of LSTM, also called a memory cell, contains two streams of input: the sequence comparison information in a sliding window and the output of the previous LSTM cell. Then, the output streams are conducted by the input, forget, and output gates responsible for updating and outputting the cell state. The input gate controls how much new information can flow into the unit. The forget gate determines how much stored information will be kept in the unit. Then, the cell status is updated by coordination of the input gate and the forget gate as given in

(6) $$\begin{array}{c} f_{t}=\sigma \left(W_{f}x_{t}+U_{f}h_{t-1}+B_{f}\right), \end{array}$$

(7) $$\begin{array}{c} i_{t}=\sigma \left(W_{i}x_{t}+U_{i}h_{t-1}+B_{i}\right), \end{array}$$

(8) $$\begin{array}{c} c_{t}=f_{t}⊗c_{t-1}+i_{t}⊗\tanh\left(W_{c}x_{t}+U_{c}h_{t-1}+B_{c}\right), \end{array}$$

(9) $$\begin{array}{c} o_{t}=\sigma \left(W_{o}x_{t}+U_{o}h_{t-1}+B_{o}\right), \end{array}$$

(10) $$\begin{array}{c} h_{t}=o_{t}⊗\tanh\left(c_{t}\right), \end{array}$$

where *f*_*t*_, *i*_*t*_, and *o*_*t*_ are the activation of the forget gate, input gate, and output gate, respectively, ⊗ denotes the element-wise multiplication, *σ* is the logistic sigmoid function, tanh is the tanh function to force the values to be between -1 and 1, *W*_*f*_, *W*_*i*_, *W*_*c*_, *W*_*o*_, *U*_*f*_, *U*_*i*_, *U*_*c*_, and *U*_*o*_ are weight coefficients, and *B*_*f*_, *B*_*i*_, *B*_*c*_, a6nd *B*_*o*_ are bias coefficients. Taking a stream {*x*_*t*_, *h*_*t*−1_} as the input, the LSTM units have the hidden states {*h*} and cell states {*c*} and each unit outputs a sequence {*o*}.

The information of the BLSTM layer is obtained by the forward ${\overset{⟶}{h}}_{t}$ and backward hidden states ${\overset{⟵}{h}}_{t}$, which link the data sequences in two separate hidden layers (equations (11)–(13)), respectively:

(11) $$\begin{array}{c} {\overset{⟶}{h}}_{t}=\text{Bl}\left(h_{t},{\overset{⟶}{h}}_{t-1}\right), \end{array}$$

(12) $$\begin{array}{c} {\overset{⟵}{h}}_{t}=\text{Bl}\left(h_{t},{\overset{⟵}{h}}_{t-1}\right), \end{array}$$

(13) $$\begin{array}{c} h_{t}=\left({\overset{⟶}{h}}_{t},{\overset{⟵}{h}}_{t}\right), \end{array}$$

where Bl is a bidirectional recurrent neural function.

#### 2.3.3. Fully Connected Layer

The vector *H* = [*h*^1^, ⋯, *h*^*T*^] responsible for processing features of the paired sequence is passed through a fully connected hidden layer with *fc* hidden units, and this process is described by

(14) $$\begin{array}{c} Fc=F\left({HW}_{fc}\right), \end{array}$$

where *F* is the ReLU function and *W*_*fc*_ is the weight coefficient.

#### 2.3.4. Sigmoid Decision Unit

Finally, the decision unit gives a score between 0 and 1, as illustrated by

(15) $$\begin{array}{c} P\left(y=1\;|\;x\right)=\frac{1}{1+\exp\left(-{FcW}_{a}\right)},\;P\left(y=0\;|\;x\right)=1-P\left(y=1\;|\;x\right), \end{array}$$

where *W*_*a*_ represents the final output weight matrix.

### 2.4. Evaluation of Performance

Agreement, a measure of how close the prediction results are to the experimental results, is defined by the following equation:

(16) $$\begin{array}{c} \text{agreement}=\frac{\text{tp}+\text{tn}}{\text{tp}+\text{tn}+\text{fp}+\text{fn}}, \end{array}$$

where tp is the number of true positives (correctly predicted antigenic variation), fn is the number of false negatives (incorrectly predicted antigenic similarity), tn is the number of true negatives (correctly predicted antigenic similarity), and fp is the number of false positives (incorrectly predicted antigenic variation).

Sensitivity, which is the ability to identify true antigenic variation correctly, is defined by

(17) $$\begin{array}{c} \text{sensitivity}=\frac{\text{tp}}{\text{tp}+\text{fn}}. \end{array}$$

Specificity, which is the ability to identify true antigenic similarity correctly, is defined by

(18) $$\begin{array}{c} \text{specificity}=\frac{\text{tn}}{\text{tn}+\text{fp}}. \end{array}$$

The Matthews correlation coefficient (MCC) [44], which takes into account true and false positives and negatives, is generally considered a balanced measure of the performance of a prediction model on the validation set. MCC is defined by

(19) $$\begin{array}{c} \text{MCC}=\frac{\text{tp}\times \text{tn}-\text{fp}\times \text{fn}}{\sqrt{\left(\text{tp}+\text{fp}\right)\times \left(\text{tp}+\text{fn}\right)\times \left(\text{tn}+\text{fp}\right)\times \left(\text{tn}+\text{fn}\right)}}. \end{array}$$

Essentially, MCC is a correlation coefficient between the observed and predicted binary classifications, with the values of 1, 0, and −1 indicating a perfect agreement, no better than random prediction, and total disagreement between prediction and observation, respectively.

## 3. Results

### 3.1. Hyperparameter Tuning

Our deep learning approach for antigenic variation prediction contains some hyperparameters, which should be tuned to achieve good performance. As seen from Table 1, in the deep learning approach, Convolution1D layers contain different filter numbers and kernel sizes and BLSTM layers contain different numbers of memory cells, while other parameters were set to fixed values. At first, we explored for an optimal combination of filter numbers (8, 16, 32, and 64) and kernel sizes (2, 5, 10, and 15) when the number of memory cells was set to a fixed value of 128. The results (Table S2) show that although the combination of filter number of 32 and kernel size of 10 has the best prediction effect (i.e., the highest MCC value, 0.960), the model with the kernel size of 15 obtains better and more stable prediction effects regardless of the filter number. It appears that the change in the filter number has an uncertain impact on the prediction effect (Table S2). Therefore, we set the kernel size to a fixed value of 15 while tuning the hyperparameters of the filter number and memory cell number to optimize the prediction effect. The results (Table S3) reveal a trend of improved prediction effect with increased filter number, and in particular, the filter number of 64 gives the best prediction effect regardless of the memory cell number. Furthermore, it appears that the too large (256) and too small (32) numbers of memory cells are not conducive to the prediction effect; however, the approach achieves stable and excellent prediction effects with 128 memory cells (Table S3). To this end, in our deep learning approach, the parameters of the kernel size, filter number, and memory cell number were set to 15, 64, and 128, respectively, for the final prediction.

### 3.2. Feature Selection

It is well known that the antigenic phenotype of flu viruses is determined by both the sequence and structural features of the HA protein; nevertheless, the extracted structural features can be mapped to the protein primary sequence through appropriate coding and, hence, can be tested by the deep learning approach. Since it appears infeasible to test all the features potentially involved in the viral antigenicity, here only four key features accounting for antigenic variation were tested using the current deep learning framework, i.e., position (HA1 sequence position where mutation occurs), epitope (whether a residue resides on the antibody-specific epitopes or not), RBD (whether a residue belongs to RBD or not), and Gly (whether a residue is at the glycosylation site or not).

Since mutation plays a crucial role in altering antigenicity, the position feature was used in all models, and the other three features were used as auxiliary information to improve the prediction performance. Here, four deep learning models with position feature alone and in combination with each of the other three features were tested to select the best prediction model. As shown in Table 2, the model using only the position feature (position model) provides good prediction results in terms of the agreement, sensitivity, specificity, and MCC. Compared to the position model, the model with the additional epitope feature (position-epitope model) notably improves the prediction results, with agreement, sensitivity, specificity, and MCC reaching up to 97.16%, 96.85%, 97.34%, and 0.939, respectively. Surprisingly, the models with additional features of Gly (position-Gly) and RBD (position-RBD) produced the results no better than those from the position model (with the exception of specificity by the position-RBD model). Therefore, only the two features, position and epitope, were incorporated into the deep learning approach for the subsequent antigenic variation prediction.

### 3.3. Performance

The performance of our deep learning approach can be evaluated by comparing its prediction results with those of the other existing methods. Since the existing methods and our deep learning approach were trained on quantitative antigenic distances and qualitative antigenic characters, respectively, it is unreasonable to perform direct comparisons between the prediction results produced through different training sets; this is circumvented by comparing the prediction results obtained from the same or similar validation set(s), regardless of the difference between the training sets. Of note is that the four machine learning methods, i.e., Multiple regression [18], Multiple regression on physicochemical properties [20], Decision tree [19], and Joint random forest method [23] (see Table 3), collectively used the complete Smith's dataset [36] as the validation set. Since this dataset contains abundant virus pairs in which there are more than nine residue mutations capable of causing antigenic variations with a probability of 99%, the prediction results by these methods show relatively high agreements and sensitivities. However, the prediction specificities of the two methods, multiple regression on physicochemical properties and joint random forest, are very low, thus leading to low MCC values. In order to avoid inflating the prediction effect, Tan et al. [32] constructed a concise dataset through removing from Smith's dataset the virus pairs with more than nine antigenic variation-causing mutations and further the redundant pairs. Although the concise dataset makes it more realistic and challenging for the prediction of changes in the antigenicity, the application of the stacked autoencoder (SAE) model, a deep learning method developed by Tan et al. [32], to the concise dataset achieved a considerably improved specificity (93%) compared to those by the two models, multiple regression on physicochemical properties (82.30%) and joint random forest (77.7%). Encouragingly, the application of our deep learning approach to our more concise dataset (see Section 2.1) further improves the prediction specificity (97.34%) relative to that by SAE. Moreover, among all the existing methods/models listed in Table 3, our deep learning approach also achieves the best prediction performance in terms of the agreement and MCC, indicating that our approach is far superior to the others and is suitable for the sequence-based prediction of antigenic variation.

The difference in antigenic properties between the circulating flu viruses and the strains prevalent in previous seasons provides the evidence basis for selecting flu vaccine strains. Thus, an effective approach capable of predicting the vaccine strains should correctly predict, on the basis of historical training data, the antigenic profile of the strains that will circulate in the upcoming season. Here, the accuracy of our deep learning approach for predicting antigenic variation of the strains in the following year was evaluated by using the historical training data. As shown in Table 4, our deep learning approach achieves an average agreement of 99.20% for predicting antigenic variation of the strains in the forthcoming year. Such excellent prediction accuracy is significantly higher than that by the Antigen-Bridges method [21] using different residue sets (Table 5). Although the agreement value of our approach decreases to 96.46% for the strains in the next two years, it is still much higher than that of the Antigen-Bridges method. In addition, our approach shows a smaller reduction in the prediction accuracy for the strains between the forthcoming year and the next two years than that of the Antigen-Bridges method (Table 5). Finally, when taking a comprehensive look at the performance measures, it can be found that our deep learning approach also achieves both the high sensitivities and specificities for the strains in the next year and next two years; furthermore, a better balance between the sensitivity and specificity for the prediction of the next-year strains than for that of the next-two-year strains leads to a higher MCC value for the prediction of the next-year strains (Table 4).

## 4. Discussion

In the current study, we incorporated the information on amino acid residue changes and several other features associated with antigenicity into a deep learning framework to predict the antigenic variation of flu A H3N2. Due to the deep combination of CNN and BLSTM, it can be expected that our deep learning approach has the capacity to capture and process both the local and nonlocal information. Indeed, our deep learning approach achieves very competitive prediction results in terms of the agreement, sensitivity, specificity, and MCC on the subset of a stricter and more concise Smith's dataset, respectively (Table 3). More encouragingly, based on the existing set of chronological amino acid sequences, our deep learning approach achieves 99.20% of agreement for antigenicity prediction of the strains in the forthcoming year and at the same time improves the sensitivity and specificity to 98.59% and 99.32%, respectively (Table 4). When compared to previous studies [21, 23], our approach improves or maintains the specificity without impairing the sensitivity, thus leading to a very high performance score of MCC (0.972) for the strains in the forthcoming year. As for the strains in the validation sets of the next two years, our approach obtains a relatively low MCC value (0.830) due to the slightly impaired balance between the sensitivity and specificity; nevertheless, our approach still offers excellent performance in terms of the agreement, sensitivity, and specificity (Table 4).

The results of hyperparameter tuning show that our deep learning approach gains the most optimal performance using the kernel size of 15 in Convolution1D layers and memory cell number of 128 in BLSTM layers (Tables S2 and S3). The kernel size of 15 means that the CNN is able to capture more local complex features in protein sequences using 15 adjacent amino acid residues than using fewer residues. The memory cell number of 128 means that the long-distance dependency encoding module is able to learn more long-distance dependency based on the local features (captured by the local feature encoding module) when the LSTM output dimension is 128. In general, more memory cells imply that more information will be extracted and learned from a complete sequence pair. Indeed, there is a trend of increasing the performance of our approach as the number of memory cells increases from 32 to 128, and a similar trend was also observed in a previous study aimed at improving the accuracy of protein secondary structure prediction with a hybrid deep learning framework [45]. However, when the number of memory cells increases to 256, the performance of our deep learning approach becomes slightly worse and more unstable compared to that with the memory cell number of 128, and this may be due to the difficulty in convergence arising from too many parameters. Previous studies have also shown that the deep learning frameworks with moderate numbers of BLSTM memory cells, 50-150, were able to achieve optimal performance in their respective prediction applications [42, 45–47].

The deep learning approach has a huge advantage in processing large amounts of complex information. In general, the more information the training set can provide, the better prediction performance the deep learning model will achieve. In this work, in addition to the sequence-based position feature, information on several structure-derived features involved in antigenic variation was also encoded and tested using deep learning models with different combinations of the position feature and each of the other features (i.e., epitope, RBD, and Gly), and the results show that only in the case of the combined position and epitope features can the model achieve the best prediction performance among all the models (Table 2). It is speculated that this may be due to the limitations of our feature extraction method and of the qualitative set of quasi-experimental HI data. A further study is needed to examine the effects of the combinations of more than two features on the prediction performance of our deep learning approach. It should also be noted that the HI experimental data of pairwise viruses are currently very limited, and therefore, we only tested the prediction effect of our deep learning approach using the quasi-experimental HI data in both the training and validation sets. We anticipate that the accuracy and performance of our deep learning method will be further improved if adequate high-quality data of HI assays are available.

## Figures and Tables

**Table 1:** The main layers and their optional parameters in the deep learning approach.

Layer	Parameter
Convolution1D_1	Filter (8, 16, 32, and 64), kernel size (2, 5, 10, and 15), strides (1)
MaxPooling_1	Pool size (2), strides (1)
Convolution1D_2	Filter (8, 16, 32, and 64), kernel size (2, 5, 10, and 15), strides (1)
MaxPooling_2	Pool size (2), strides (1)
BLSTM_1	Memory cell (32, 64, 128, and 256)
BLSTM_2	Memory cell (32, 64, 128, and 256)
Dense_1	Output space (64)
Dropout_1	Rate (0.6)
Dense_2	Output space (25)
Dropout_2	Rate (0.6)
Softmax	Output space (1)

**Table 2:** The prediction results obtained from different deep learning models with the position feature alone and in combination with the other three features.

Model	Agreement (%)	Sensitivity (%)	Specificity (%)	MCC
Position	95.73	95.18	96.12	0.914
Position-epitope	97.16	96.85	97.34	0.939
Position-Gly	95.02	93.84	95.75	0.895
Position-RBD	94.74	92.42	96.44	0.892

**Table 3:** The prediction performance of our deep learning approach and other existing approaches.

Approaches	Training set	Validation set	Agreement^a^ (%)	Sensitivity^a^ (%)	Specificity^a^ (%)	MCC^a^
Multiple regression [18]	181 HI experiments	31878 pairs in Smith's dataset^b^	89.89	—	—	—
Multiple regression on physicochemical properties [20]	394 HI experiments	31878 pairs in Smith's dataset^b^	96.96	99.55	82.30	0.877
Decision tree [19]	181 HI experiments	31878 pairs in Smith's dataset^b^	96.20	—	—	—
Joint random forest method^c^ [23]	28690 pairs in Smith's dataset	31878 pairs in Smith's dataset^b^	96.4	98.1	77.7	0.758
Stacked autoencoder^d^ [32]	80% of the 8097 pairs in a concise version of Smith's dataset	20% of the 8097 pairs in a concise version of Smith's dataset	95	95	93	—
Our deep learning approach^e^	The filtered virus pairs formed by 70% of 253 strains in Smith's dataset	The filtered virus pairs formed by 30% of 253 strains in Smith's dataset	97.16	96.85	97.34	0.939

^a^The mark “—“ means that there is no relevant data in literature. ^b^Smith's dataset contains 31878 pairwise comparisons among 253 viral strains that belong to 11 clusters; out of the 31878 virus pairs, 27098 pairs composed of the strains from different clusters contain antigenic variations, whereas 4780 pairs composed of the strains from the same clusters possess similar antigens [36]. ^c^Yao et al. performed 10-fold cross-validation on Smith's dataset. ^d^The stacked autoencoder model was developed based on a concise dataset obtained by removing from Smith's dataset the sequence pairs that contain more than 9 antigenic variation-causing mutations followed by further removing the redundant pairs. ^e^Our deep learning method was developed based on a more concise dataset built from Smith's dataset (for details of constructing the dataset, see Section 2.1); the advantage of our dataset is that the virus pair-constituting strains in the training set and validation set are completely nonoverlapping or different.

**Table 4:** The results of the antigenic variation prediction for flu A H3N2 in the forthcoming year and in the next two years using our deep learning approach.

Prediction duration	Agreement (%)	Sensitivity (%)	Specificity (%)	MCC
Next year	99.20	98.59	99.32	0.972
Next two years	96.46	98.58	96.24	0.830

**Table 5:** Comparison between the agreements obtained by our deep learning approach and the Antigen-Bridges method with three residue sets [21] for the strains in the forthcoming year and in the next two years.

Approaches (amino acid number)	Next year (%)	Next two years (%)
Antigen-Bridges (39-residue set)	83.78	75.10
Antigen-Bridges (44-residue set)	79.75	72.48
Antigen-Bridges (25-residue set)	80.51	71.51
Our deep learning approach	99.20	96.46

## Data Availability

Research data can be obtained by contacting the first author or corresponding author: Yuan-Ling Xia (xiayl@ynu.edu.cn) or Shu-Qun Liu (shuqunliu@ynu.edu.cn).

## Supplementary Materials

Supplementary Materials Table S1: The one-hot coding for 20 amino acids. Table S2: The MCC obtained from our deep learning approach with different combinations of the kernel size and filter number in Convolution1D layers, and the number of memory cells in LSTM being set to 128. Table S3: The MCC obtained from our deep learning approach with different combinations of the filter number in Convolution1D layers, and the number of memory cells in LSTM and the kernel size of Convolution1D layers being set to 15.
