# Supplementary material for: A Deep Learning Approach for Predicting Antigenic Variation of Influenza A H3N2
Source: Comput Math Methods Med. 2021 Oct 16;2021:9997669. doi: 10.1155/2021/9997669 (PMC8541863; doi:10.1155/2021/9997669)
Supplement: Supplementary Materials — Table S1: The one-hot coding for 20 amino acids. Table S2: The MCC obtained from our deep learning approach with different combinations of the kernel size and filter number in Convolution1D layers, and the number of memory cells in LSTM being set to 128. Table S3: The MCC obtained from our deep learning approach with different combinations of the filter number in Convolution1D layers, and the number of memory cells in LSTM and the kernel size of Convolution1D layers being set to 15. [file 9997669.f1.docx]

**Supporting Information**

Table S1 The one-hot coding for 20 amino acids.

| Amino acid | One-hot coding |
| --- | --- |
| Gly | 10000000000000000000 |
| Ala | 01000000000000000000 |
| Val | 00100000000000000000 |
| Leu | 00010000000000000000 |
| Ile | 00001000000000000000 |
| Phe | 00000100000000000000 |
| Pro | 00000010000000000000 |
| Trp | 00000001000000000000 |
| Ser | 00000000100000000000 |
| Tyr | 00000000010000000000 |
| Cys | 00000000001000000000 |
| Met | 00000000000100000000 |
| Asp | 00000000000010000000 |
| Asn | 00000000000001000000 |
| Glu | 00000000000000100000 |
| Gln | 00000000000000010000 |
| Thr | 00000000000000001000 |
| Lys | 00000000000000000100 |
| Arg | 00000000000000000010 |
| His | 00000000000000000001 |

Table S2 The MCC obtained from our deep learning approach with different combinations of kernel size and filter number in Convolution1D layers, and the number of memory cells in LSTM being set to 128.

| Kernel size | Filter number | | | |
| --- | --- | --- | --- | --- |
|  | 8 | 16 | 32 | 64 |
| 2 | 0.926 | 0.874 | 0.930 | 0.935 |
| 5 | 0.925 | 0.920 | 0.929 | 0.930 |
| 10 | 0.924 | 0.921 | 0.960 | 0.922 |
| 15 | 0.938 | 0.939 | 0.935 | 0.937 |

Table S3 The MCC obtained from our deep learning approach with different combinations of filter number in Convolution1D layers and the number of memory cells in LSTM, and the kernel size of Convolution1D layers being set to 15.

| The memory cells of LSTM | Filter number | | | |
| --- | --- | --- | --- | --- |
|  | 8 | 16 | 32 | 64 |
| 32 | 0.909 | 0.908 | 0.921 | 0.939 |
| 64 | 0.911 | 0.918 | 0.929 | 0.947 |
| 128 | 0.931 | 0.947 | 0.934 | 0.944 |
| 256 | 0.922 | 0.929 | 0.930 | 0.939 |
